# Remarks upon the Treatment of Pneumonia

**Published:** 1881-06-20

**Authors:** R. C. Word

**Affiliations:** Professor of Physiology in the Southern Medical College


### Southern Medical Record:

EDITORS:

T. S. POWELL, M.D. W. T. GOLDSMITH, M.D. R. C. WORD, M.D.

*R. C. WORD, M.D., Managing Editor.*

☞All Communications and Letters on Business connected with the RECORD must be addressed to the Managing Editor.

VOL. XI. ATLANTA, GA., JUNE 20, 1881. No. 6.

### ORIGINAL AND SELECTED ARTICLES.

### REMARKS UPON THE TREATMENT OF PNEUMONIA.

BY R. C. WORD, M.D.,

Professor of Physiology in the Southern Medical College.

A correspondent calls for our views in regard to the treatment of pneumonia. We published in our April issue an article by Dr. E. P. Townsend, which so nearly corresponds with our own views and experience, that we are thereby spared the labor of a special paper on the subject.

We rely in a great measure upon veratrum, aconite and opiates in the treatment of pneumonia. We have found that opiates counteract the nauseating tendency of veratrum, without preventing its controling influence upon the circulation. We prefer the muriate of morphia and McMunn's elixir to other forms of opium. We usually commence the veratrum with doses of two or three drops at intervals of two hours, increasing a drop with each dose until the pulse is brought down to near its normal standard; and then we continue the remedy at intervals of three hours at the same or in reduced doses, according to the condition of the pulse.

We do not give quinine in conjunction with veratrum except in cases where there are exascerbations of fever with a high temperature, in which case quinine internally, or used externally with whisky upon the skin, will bring down the temperature and assist greatly in controling the fever. In cases of asthenic type, there is danger of too great depression from the use of quinine and veratrum at the same time. In most cases the veratrum alone will sufficiently control both the circulation and temperature.

We use mustard and poultices to the chest, and in some cases blisters are applied to the affected side. Aconite agrees well with some cases, and may be substituted for the veratrum where, from any cause, the latter article may not suit the particular case, or where the sweating and softness of pulse indicate that the remedy is sufficiently pushed; here the aconite, with the carbonate of ammonia, as in the article above referred to, will usually act well. Or—

| | | |
|---|---|---|
| ℞ | Carb. ammonia.................................... | ʒi. |
| | Water................................................ | ℥iv. |
| | Tinct. aconite radicis........................ .... | gtts. x. |

S. A teaspoonful every one to two hours.

If the depression be great and the aconite doubtful, the carbonate of ammonia, with a little morphia and the infusion of serpentaria, will supply the indication, assisting the expectoration and supporting the patient:

| | | |
|---|---|---|
| ℞ | Carb. ammonia.................................. | ʒi. |
| | Infusion serpentaria............................ | ℥iv. |
| | Muriate morphia.................................. | gr. i. |

Dose, teaspoonful every two to three hours.

This preparation answers well when a stimulating expectorant is indicated. The ammonia and serpentaria soften the sputa and promote its easy expectoration, and serve to counteract the drying or astringent tendency of the morphine, which nevertheless exerts its anodyne influence in keeping down the pain and promoting the comfort of the patient.

It should not be inferred from the above remarks that veratrum is necessarily contra-indicated in the asthenic forms of disease. Its cautious administration, especially when combined with opiates and revulsives, and even stimulants, is often very effectual in such cases. It will generally be well tolerated when there is a full pulse.

In a recent case which came under our observation, the patient having but a short while before recovered from measles, the symptoms indicated a low or asthenic tendency—so much so, that it was not deemed prudent to give veratrum. Quinine, Dover's powder and aconite, with revulsives, etc., were used for some days, until the pulse, gradually increasing, had reached 138, with other dangerous indications. Yet the pulse, though rapid, was full. It was now decided to try the veratrum. At 7 o'clock P.M, three drops were given, and the dose repeated every hour and a half, increasing a drop with each dose. At the third or fourth dose an impression was made upon the circulation; the pulse, though falling in frequency, retained its volume, and by 12 o'clock was below 100, and before morning fell to 80. The remedy was continued, the circulation kept down and the patient saved!

Pneumonia may be aborted if taken promptly in the early stage or during the initial rigor, and in some instances even after the reaction and pain in the chest is well developed. If you are so fortunate as to see the patient at this early period, give at once quinine and Dover's powder, each ten grains, and apply a large sinapism to the chest—and, if the patient be chilly, hot rocks to the feet. If the pain is not quickly relieved, repeat the opiate. The result is usually prompt relief of pain, a short febrile stage, followed by sleep, perspiration, and a rapid recovery of the patient.

If in a well-developed case of pneumonia we were deprived of the use of veratrum and aconite, as in the method we have described, we should resort to blood-letting, followed by quinine, revulsives, opiates and minute doses of tartar emetic.

